# Variability in the validity and reliability of outcome measures identified in a systematic review to assess treatment efficacy of cognitive enhancers for Alzheimer’s Dementia

**DOI:** 10.1371/journal.pone.0215225

**Published:** 2019-04-18

**Authors:** Charlene Soobiah, Mina Tadrous, Sandra Knowles, Erik Blondal, Huda M. Ashoor, Marco Ghassemi, Paul A. Khan, Joanne Ho, Andrea C. Tricco, Sharon E. Straus

**Affiliations:** 1 Institute for Health Policy, Management & Evaluation, University of Toronto, Toronto, Ontario Canada; 2 Li Ka Shing Knowledge Institute of St. Michael’s Hospital, Toronto, Ontario Canada; 3 Leslie Dan Faculty of Pharmacy, University of Toronto, Toronto, Ontario Canada; 4 Clinical Pharmacology & Toxicology, Department of Pharmacy, Sunnybrook Health Sciences Centre, Toronto, Ontario Canada; 5 Schlegel Research Institute for Aging, Waterloo, Ontario Canada; 6 Department of Medicine, McMaster University, Hamilton, Ontario Canada; 7 Epidemiology Division, Dalla Lana School of Public Health, University of Toronto, Toronto, Ontario Canada; 8 Division of Geriatric Medicine, Department of Medicine, University of Toronto, Suite RFE 3–805, Toronto, Ontario Canada; Nathan S Kline Institute, UNITED STATES

## Abstract

**Introduction:**

Selection of optimal outcome measures is a critical step in a systematic review; inclusion of uncommon or non-validated outcome measures can impact the uptake of systematic review findings. Our goals were to identify the validity and reliability of outcome measures used in primary studies to assess cognition, function, behaviour and global status; and, to use these data to select outcomes for a systematic review (SR) on treatment efficacy of cognitive enhancers for Alzheimer’s Dementia (AD).

**Methods:**

Articles fulfilling the eligibility criteria of the SR were included in a charting exercise to catalogue outcome measures reported. Outcome measures were then assessed for validity and reliability. Two independent reviewers abstracted data on outcome measures and validity and reliability reported for cognition, function, behaviour and global status.

**Results:**

129 studies were included in the charting exercise; 57 outcome measures were identified for cognition, 21 for function, 13 for behaviour and 10 for global status. A total of 35 (61%) cognition measures, 10 (48%) functional measures, 8 (61%) behavioural measures and four (40%) of global status measures were only used once in the literature. Validity and reliability information was found for 51% of cognition measures, 90% of function and global status measures and 100% of behavioural measures.

**Conclusions:**

While a large number of outcome measures were used in primary studies, many of these were used only once. Reporting of validity and reliability varied in AD studies of cognitive enhancers. Core outcome sets should be used when available; when they are not available researchers need to balance frequency of reported outcome measures, their respective validity and reliability, and preferences of knowledge users.

**Systematic review registration:**

CRD#42012001948

## Introduction

Outcome measures are tools, instruments or scales used to assess an outcome. For example, the Activities of Daily Living (ADL) [1, 2] is to assess function in older adults. Selection of appropriate outcome measures for inclusion in a systematic review is imperative, to ensure research relevance for knowledge users [3]. Knowledge users are individuals who may use research findings to make a decision and can include patients, clinicians, policymakers, or researchers [4, 5]. Inclusion of non-validated or uncommon measures in a systematic review can make it difficult for knowledge users to interpret and utilize findings to make informed decisions [6, 7]. Multiple measures exist for a particular outcome and their selection for use in systematic reviews can be challenging; a researcher must identify measures that are valid, reliable, and clinically relevant [8].

We previously conducted a systematic review and network meta-analysis on the comparative safety and efficacy of cognitive enhancers for treatment of Alzheimer’s Dementia (AD). This review was conducted with geriatricians and policymakers (i.e. knowledge users) who wanted to use the results to inform decision-making on medication use in Canada [9, 10]. During the systematic review process, numerous outcome measures were identified for our pre-specified outcomes (i.e., cognition, function, behaviour, and global status), as such we sought to identify the validity and reliability of outcome measures used in primary studies to assess out pre-specified outcomes; and, to use these data to select outcomes for inclusion in our systematic review.

## Methods

As our systematic review methods and results were previously published [9], the focus of this paper is on how outcome measures were identified for inclusion in the review. Our systematic review was registered (CRD#42012001948) [10] and included experimental and observational studies that reported on cognitive enhancers approved for use in Canada (i.e., donepezil, galantamine, rivastigmine and memantine) for patients with AD. Studies had to report on at least one pre-specified outcome (i.e., cognition, function, behaviour, and global status) to be considered eligible for inclusion in the systematic review. Several electronic databases were searched from inception to December 31, 2011 to identify studies. Two independent reviewers assessed each citation and full text article against eligibility criteria. Studies fulfilling eligibility criteria were included in the present study.

### Charting exercise

Primary studies fulfilling the eligibility criteria were included in a data charting exercise. For each study, we catalogued the measures that were used for our pre-specified outcomes of interest. This approach is frequently used in scoping reviews to synthesize information and understand knowledge gaps [11] This step was conducted prior to data abstraction in the systematic review -. The literature search for the systematic review was updated prior to publication; however, we used the original literature search for this current study.

A standardized spreadsheet (Excel) was created to capture the measures used to assess each outcome. A calibration exercise was conducted with reviewers (CS, ACT, JH, EB, HA, MG, PAK) on a random subset of studies (n = 10) until adequate agreement was achieved (>80% agreement) and the spreadsheet was modified accordingly. Reviewers independently abstracted outcome measures using the standardized spreadsheet.

### Validity and reliability assessment

To obtain validity and reliability information for each measure in the charting exercise a three-step process was employed. First, references of primary studies included in the systematic review were used to locate the measure citation. Second, if the measure was not cited, an electronic literature search was conducted in MEDLINE, Mental Measures Yearbook, Health and Psychosocial Instruments and/or Google Scholar (from inception to January 2015) using the measure name and keywords such as ‘validation’, ‘psychometric’ and ‘reliability’. Third, if the validity or reliability information for a measure were not located by searching, authors of the included study were contacted to request this information.

A second standardized spreadsheet was created to capture validity and reliability information and a calibration exercise was conducted with reviewers (CS, MT, SK) on a random subset of studies (n = 10) until adequate agreement was achieved (>80% agreement). The spreadsheet was modified accordingly. Next, pairs of reviewers (CS, MT, and SK) abstracted validity and reliability information independently.

Validity and reliability information were categorized using the description reported by the authors in the cited studies. When type of validity or reliability examined was not explicitly stated or was unclear, we used an established framework to categorize the data [11]. This framework suggests that all forms of validity fall under the category of construct validity, which can be further divided into translational (face or content) and criterion validity (concurrent, convergent, predictive, discriminant and predictive; S1 Table) [11]. To estimate reliability of an outcome measure, internal consistency, test-retest and inter-rater reliability were used (S1 Table) [11]. Funding was categorized as industry sponsored (e.g., funding received from the private sector), mixed funding (e.g., funding derived from private and public sectors) and non-industry sponsored (e.g., funding from the public sector).

## Analysis

Data from the charting exercise and validity and reliability assessments were analyzed descriptively using frequencies in Excel.

## Results

### Charting results

In total, 15,556 citations were screened, of which 129 full-text articles were included in the charting exercise. Overall, 101 unique outcome measures were identified including: 57 measures for cognition, 21 for function, 13 for behaviour, and 10 for global status (S2, S3, S4 and S5 Tables).

### Frequency of outcome measures

For cognition assessment, the most frequent outcome measures identified from our literature search were the: Mini-Mental State Exam (MMSE)[12] reported in 80 studies; Alzheimer’s Disease Assessment Scale–cognitive subscale (ADAS-cog) [13] reported in 61 studies; and Severe Impairment Battery (SIB)[14] reported in 13 studies (S2 Table). Only 7 (12%) measures were used in more than 5 primary studies. A total of 35 (61%) cognition measures were used once.

For function assessment, the most frequently used outcome measures were: Activities of Daily Living (ADL) [2] reported in 19 studies, Alzheimer’s disease Cooperative Studies–ADL (ADAS-ADL)[15] and Disability Assessment for Dementia (DAD)[16, 17] reported in 7 studies (S3 Table). Ten (48%) functional measures were used once.

For behavioral assessment, the most frequently reported outcome measures were the: Neuropsychiatric Inventory (NPI) [18] reported in 36 studies; Behavioural Pathology in Alzheimer’s Disease Rating Scale (BEHAVE-AD)[19, 20]; and, Cohen-Mansfield agitation Inventory (CMAI)[21] reported in 5 studies (S4 Table). Eight (61%) behavioural measures were used once.

For global status, the most frequently reported outcome measures were the Clinician Interview-Based Impression of Change plus caregiver input (CIBIC-plus)[22] reported in 35 studies, and the Clinical Global Impression of Change (CGIC)[23] reported in 10 studies (S5 Table). Four (40%) global status measures were used once.

### Reporting of validity and reliability of outcome measures

**Fig 1:**
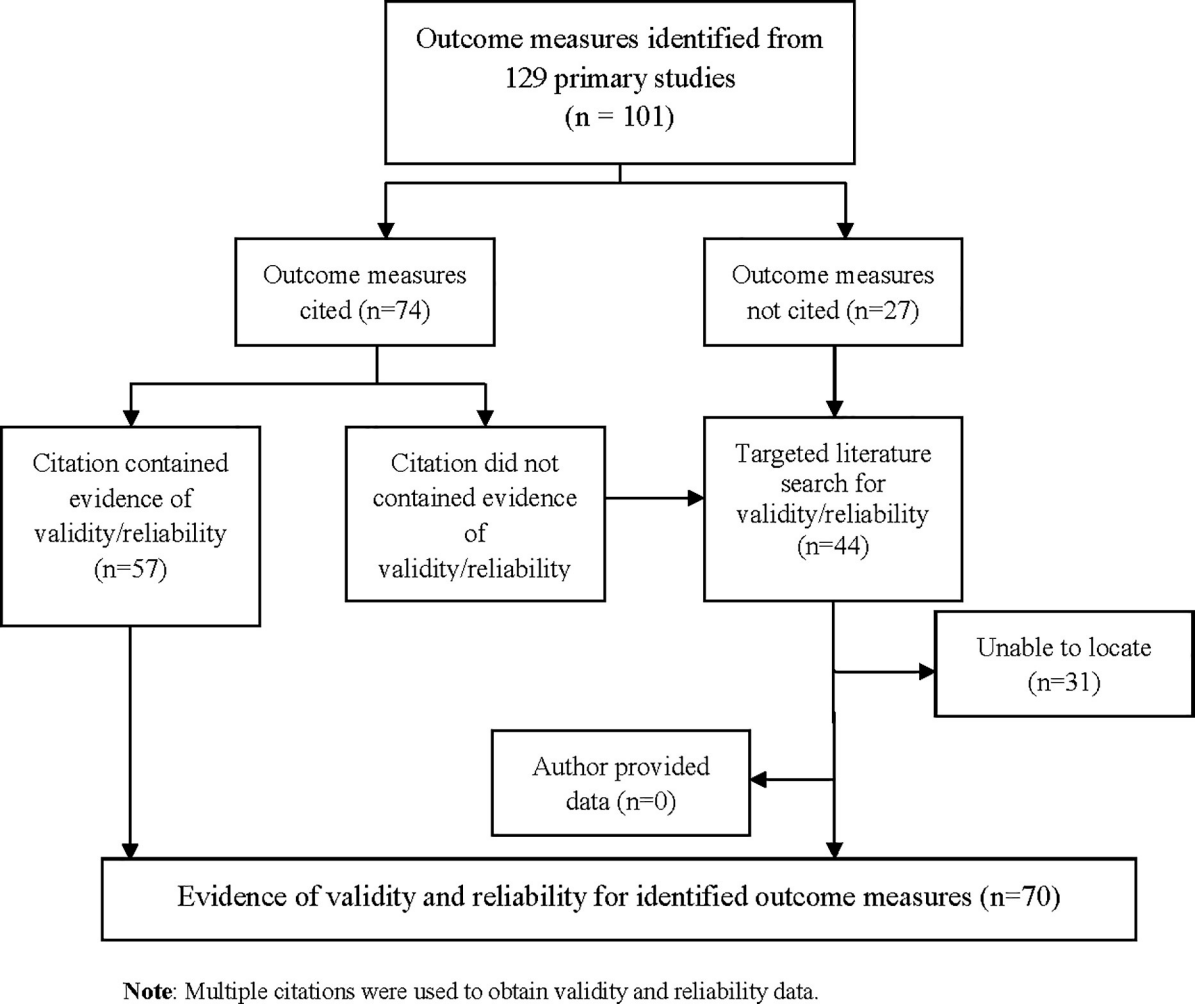
Flow diagram of locating validity and reliability.

Of the 101 outcome measures identified from the 129 primary studies, 74 (73%) outcome measures were supported by citation of references while 27 (27%) outcome measures were not (Fig 1). Of the 74 outcome measures that had citations, 57 (77%) citations reported evidence of validity and/or reliability (i.e., 22 for cognitive measures, 17 for functional measures, 11 for behavioural measures, 7 for global measures) (Fig 1). The citations for the remaining 17 outcome measures (23%) did not contain validity or reliability information or the source was irretrievable. Citations for these 17 outcome measures included 8 textbooks, 8 journal articles and 4 test manuals. We were unable to access the cited test manuals for Digit Span, Digit Symbols Test, Category Fluency Test, and the Wechsler Adult Intelligence Scale, as the material was proprietary. The citation identified for the MENFIS outcome measure, led to a non-English article [24] and translation was not possible.

We conducted a targeted literature search for 44 outcome measures that were not supported by appropriate citations in the primary studies; these included 35 cognitive measures, 4 functional measures, 3 global measures and 2 behavioural measures. Validity and reliability could not be located for 31 (31%) outcome measures, the majority (n = 28) of which addressed cognition (Fig 1). Six study authors were contacted; however validity or reliability data were not provided.

Validity and reliability data could not be located for 31 outcome measures that were reported in 20 primary studies. Of these 20 studies, 7 (35%) studies were funded by a mix of industry and non-industry sponsors, 6 (30%) were funded by industry sponsors, and, 2 (10%) were funded by non-industry sponsors. Five (25%) studies did not disclose funding (S6 Table).

Overall, validity and reliability information was located for 51% of cognitive measures (n = 29), 90% of functional measures (n = 10) and 90% global status measures (n = 9). Validity or reliability data were available for all behavioural outcome measures (n = 13).

### Cognition outcome measures

Validity and reliability information was identified for 29 of 57 (51%) cognition measures (Table 1). Concurrent and convergent were the most frequently reported forms of validity, while content or face validity were the least reported forms.

**Table 1:** Validity and reliability of cognitive measures used in treatment efficacy studies of Alzheimer’s Dementia (n = 29).

Name of Scale (YR)	Validity						Reliability		
	Face/Content	Construct	Concurrent	Predictive	Convergent	Discriminant	Internal consistency	Test-retest	Inter-rater
ADAS (1984)[13]			✓					✓	✓
ADAS-Cog (1984)[13]			✓					✓	✓
ADAS-OT (1984)[13]							✓		
ASHA-FACS (2008)[39]			✓				✓		✓
BDS (1968)[40]				✓					
CAMCOG (1986)[41]					✓				✓
CDR (1988)[42]					✓		✓		✓
CDR-SB (1988)[43, 44]					✓		✓		✓
CDT (1989)[45]					✓			✓	
CERAD-cog (1989)[46]		✓						✓	✓
COWAT (1996)[33]							✓	✓	
DWRT (1989)[36]				✓				✓	
FAB (2000)[25]			✓			✓	✓		✓
FLCI (1994)[34, 35]								✓	
GDS (1982)[26]				✓	✓				
GMLT (2008)[27, 28]		✓			✓				
MMSE (1975)[12]			✓					✓	✓
RAVLT (1988)[47]			✓						
RCM (1989)[36]								✓	
ROCF-copy (1990)[29–31]		✓		✓			✓		✓
ROCF-recall (1990)[31]					✓			✓	✓
SIB (1997)[14]			✓					✓	
SKT (1992)[48]		✓							
SR (2011)[49]			✓						
TMT (1958)[50, 51]						✓	✓		
TMT-A (1958)[51]						✓			
WLMT (1993) [37, 38]								✓	✓
WMS (1990)[32]		✓			✓				
ZVT (1985)[52]					✓				

**Abbreviations:** ADAS Alzheimer’s Disease Assessment Scale; ADAS-Cog Alzheimer’s Disease Assessment Scale- Cognitive subscale; ADAS-OT Alzheimer’s Disease Assessment Scale—Orientation Test; ASHA-FACS American Speech-Language Hearing Association- Functional Assessment of Communication Skills for Adults Basic needs and social communication subscales; BDS Blessed Dementia Scale; CAMCOG Cambridge Cognitive Examination; CDR Clinical Dementia Rating Scale; CDR-SB Clinical Dementia Rating Scale–Sum of Boxes; CDT Clock Drawing Test; CERARD-cog Consortium to Establish a Registry for Alzheimer’s Disease–cog subscale; COWAT Controlled Oral Word Association Test; DWR Delayed word recall; FAB Frontal Assessment Battery; FLCI Functional Linguistic Communication Inventory; GDS Global Deterioration Scale; GMLT Groton Maze Learning Task; MMSE Mini Mental State Exam; RAVLT Rey Auditory Verbal Learning Test; RCM Raven Colored Matrices; ROCF-recall Rey-Osterrieth complex figure recall; ROCF-copy Rey-Osterrieth complex figure copy; SIB Severe Impairment Battery; SR Story Recall; SKT Syndrome Kurtz test; TMT Trail Making Test; TMT-A Trail Making Test A; WLMT Wechsler Logical Memory Test; YR year ZVT Zahlen–Verbindungs Test. **NOTE**: Year reported is based on the earliest published paper reporting on validity or reliability for each outcome measure. Validity data for the following scales/measures could not be located: Digit Span; Digit Symbols test, Stroop Test; Stockholm Gerontology Research Center test; Verbal Fluency, Clock Recognition, Stockholm Gerontology Research Center test- D-prime value; Word Paradigm–free recall; Cambridge Automated Neuropsychiatric Test Assessment Battery; Cognitive Drug Research Test Battery; Category Fluency Test; Computerized Memory Battery Test; Forced Delayed Recognition; Immediate Visual Memory; Multiple Feature Target Cancellation; Non-Demanding Test of Visual Attention; NYU Stories Test- Delayed Recognition Subscale; Oral Production Test, Reading and Setting a Clock Test; Serial Reaction Time Task; Spatial Span; Test of Constructional Praxis; Temporal Rule Induction; Token Test, Visual Motor Gestalt Test; Wechsler Adult Intelligence Scale; Word Fluency; and Word Learning

The Frontal Assessment Battery (FAB)[25], Global Deterioration Scale (GDS)[26], Groton Maze Learning Test, (GMLT)[27, 28] the Rey-Osterrieth Complex Figure copy (ROCF-copy)[29–31] and the Wechsler Memory Scale (WMS)[32] had evidence of more than two forms of validity (Table 1). Four cognitive outcome measures did not have evidence of validity, namely the Controlled Oral Word Association Test (COWAT)[33], Functional Linguistic Communication Inventory (FLCI)[34, 35], Raven Coloured Matrices (RCM)(36), and Wechsler Logical Memory Test (WLMT) [37, 38].

Test-retest and inter-rater reliability were the most frequently reported forms of reliability for cognition measures. Eleven (38%) of the 29 cognition measures had evidence of two forms of reliability, 10 (34%) had evidence of one form of reliability and eight (27%) had no evidence of reliability (Table 1).

### Function outcome measures

Validity and reliability information were identified for 19 of the 21 (90%) function measures. Concurrent and convergent validity were the most frequently reported forms of validity. The Bristol Activities of Daily Living (BADL)[53], and the Caregiver Perceived Burden Questionnaire (CPBQ)[54] had evidence of three forms of validity. Six of the 19 (32%) measures had evidence of two forms of validity and 11 (58%) had evidence of one form of validity (Table 2).

**Table 2:** Validity and reliability of functional status measures used in treatment efficacy studies for Alzheimer’s Dementia (n = 19).

Name of Scale (YR)	Validity						Reliability		
	Face/Content	Construct	Concurrent	Predictive	Convergent	Discriminant	Internal consistency	Test-retest	Inter-rater
AAIQOL (1996)[58]	✓			✓				✓	
ADCS-ADL (1997)[15]	✓		✓					✓	
ADCS-ADL-severe (2005)[59]			✓				✓	✓	
ADFACS (2014)[60]			✓				✓		
ADL (1970)[1]			✓				✓	✓	✓
BADL (1996)[53]	✓	✓	✓					✓	
BI (1997)[61, 62]					✓				✓
CMCS (2000)[55]					✓				
CPBQ (2012)[54]	✓		✓		✓		✓	✓	
DAD (1999)[17, 63]	✓		✓				✓	✓	✓
FAST (1992)[64]			✓						✓
FRS (1989)[65]			✓			✓		✓	✓
GAFS (2006)[66]					✓				✓
GAS (1989)[67]					✓				✓
IADL (1970)[1]			✓		✓		✓	✓	✓
IDDD (1991)[68]			✓		✓		✓		
NOSGER (1991)[57]			✓				✓	✓	✓
PDS (1989)[69]	✓						✓	✓	
ZBI (1980)[70, 71]							✓		✓

**Abbreviations**: AAIQoL Activity & Affect Indicators of Quality of Life; ADCS-ADL Alzheimer’s Disease Cooperative Studies Activities of Daily Living Inventory; ADCS-ADL-severe Alzheimer’s Disease Cooperative Studies Activities of Daily Living Severe impairment subscale; ADFACS Alzheimer’s Disease Functional Assessment and Change Scale, BI Barthel Index; BADL Bristol Activities of Daily Living; CPBQ Caregiver-Perceived burden Questionnaire; CMCS Caregiver-rated Modified Crichton Scale; DAD Disability Assessment for Dementia; FAST Functional Assessment Screening Tool; FRS Functional Rating Scale; GAST Global Assessment of Functioning Scale; GAS Goal Attainment Scale; IADL Instrumental Activities of Daily Living; IDDD Interview for Deterioration in Daily living activities in Dementia; NOSGER Nurses Observation Scale for Geriatric Patients; ADL Physical Self-Maintenance/Activities of Daily Living; PDS Progressive Deterioration Scale; ZBI Zarit Burden Interview. **Note:** Year reported is based on the earliest published paper reporting on validity or reliability for each outcome measure. Validity data for MENFIS and CBQ could not be located

Test-retest was the most frequently reported measure of reliability. All function measures had evidence of at least one form of reliability with the exception of the caregiver-rated modified Crichton scale (CMCS) which did not report reliability [55]. Activities of Daily Living (ADL) [1, 56], DAD [16, 17], Instrumental Activities of Daily Living (IADL)[1] and the Nurses Observation Scale for Geriatric Patients (NOSGER)[57] each had evidence of all forms of reliability (Table 2).

### Behaviour outcome measures

Validity and reliability information were identified for all 13 behavioural outcome measures. Face/content and convergent validity were the most frequently reported forms of validity. The Behavioural Rating Scale for geriatric patients (BRS)[72, 73] and the Geriatric Depression Scale (GDS)[74] had evidence of more than three forms of validity. We were unable to find validity information for the Apathy Scale (AS) [75].

Inter-rater and test-retest were the most frequently reported forms of reliability for behavioural outcome measures. The Apathy scale (AS)[75], Behavioural Rating Scale for geriatric patients (BRS)[72, 76] and Neuropsychiatric Inventory (NPI)[18] had evidence of all forms of reliability. Three (23%) measures had two forms of reliability, whereas six (46%) had evidence of one form of reliability. We were unable to find evidence of reliability for the Neuropsychiatric Inventory nursing home version (NPI-NH)[77] (Table 3).

**Table 3:** Validity and reliability of behavioural status measures used in treatment efficacy studies in Alzheimer’s Dementia (n = 13).

Name of Scale	Validity						Reliability		
	Face/Content	Construct	Concurrent	Predictive	Convergent	Discriminant	Internal consistency	Test-retest	Inter-rater
AS (1992)[75]							✓	✓	✓
BEHAVE-AD (1990)[19, 73, 78]		✓							✓
b-NPI (2000)[79]					✓			✓	
b-PRS (1962)[80–84]	✓	✓						✓	✓
BRS (1997)[72, 76, 85]	✓	✓			✓		✓	✓	✓
CA-NPI (2004)[86]					✓			✓	
CGRS (1989)[87]	✓								✓
CMAI (1989)[21, 88]		✓							✓
GDS (1983)[74]	✓			✓	✓		✓	✓	
NPI (1994)[18]	✓		✓				✓	✓	✓
NPI-CDS (2000)[79]			✓					✓	✓
NPI-NH (2001)[77]			✓						
PAS (1995)[89]					✓				✓

**Abbreviations**: AS Apathy Scale; BEHAVE-AD Behavioural Pathology in Alzheimer’s Disease Rating Scale, BRS Behavioural Rating Scale for Geriatric Patients, b-NPI Brief Neuropsychiatric Inventory Scale; b-PRS Brief Psychiatric Rating Scale; CA-NPI Caregiver-Administered-Neuropsychiatric Inventory; CMAI Cohen-Mansfield Agitation Inventory; CGRS Crichton Geriatric Rating Scale; GDS Geriatric Depression Scale; NPI-CDS Neuropsychiatric Inventory–Caregiver distress scale; NPI Neuropsychiatric Inventory; NPI-NH Neuropsychiatric Inventory–Nursing Home version (NPI-NH); PAS Pittsburgh Agitation Scale YR year. **Note:** Year reported is based on the earliest published paper reporting on validity or reliability for each outcome measure.

### Global status outcome measures

Validity and reliability of global status measures were located for 9 of 10 (90%) global status outcome measures. Construct, convergent and discriminant validity were the most frequently reported forms of validity. Four (44%) of the nine global status measures had evidence of two forms of validity and two (22%) had one form of validity. The caregiver-rated global impression (Caregiver-rated GI)[90] and the CIBIC-plus(22) outcome measures did not have any associated validity evidence (Table 4).

**Table 4:** Validity and reliability of global status measures used in treatment efficacy studies in Alzheimer’s Dementia (n = 9).

Name of Scale	Validity							Reliability		
	Face/Content	Construct	Criterion	Concurrent	Predictive	Convergent	Discriminant	Internal consistency	Test-retest	Inter-rater
ADCS-CGI (1997)[92]	✓				✓				✓	
CRGI (1994)[90]									✓	
CGIC (1996)[23, 91]						✓	✓			
CGIC-severe (1992)[91, 93]						✓			✓	✓
CGI-I (2006)[91]						✓	✓		✓	
CIBIC- plus (1994)[22, 90]									✓	✓
CSS (1990)[94, 95]		✓		✓				✓		
GBS (1982)[96, 97]		✓		✓						✓
SCB (1991)[98]		✓					✓	✓		✓

**Abbreviation:** ADCS-CGIC Alzheimer’s Disease Cooperative Studies–Clinician Global Impressions of Change; CSS Caregiver Stress Scale; CRGI Caregiver-rated Global Impression; CGIC-severe Clinical Global Impression of Change—severe subscale; CGIC Clinical Global Impression of Change; CGI-I Clinical Global Impression of Improvement; CIBIC-plus Clinician Interview-Based Impression of Change plus caregiver input; GBS Gottfries-Bråne-Steen Scale, SCB Screen for Caregiver Burden. **Note** Year reported is based on the earliest published paper reporting on validity or reliability for each outcome measure. Patient Global Assessment (PGA) scale could not be located

Test-retest reliability was the most frequently reported form of reliability. We were unable to locate evidence of reliability for the Clinical Global Impressions of Change (CGIC) scale [23, 91].

## Discussion

Overall, 129 studies were included in the systematic review on treatment efficacy of cognitive enhancers for AD patients and from these articles, we identified 101 measures for our outcomes of interest (57 cognition measures, 21 function measures, 13 behaviour measures and 10 global status measures). We identified validity and reliability data for 51% of cognition measures, 100% of behaviour measures and 90% of function measures and global status measures. Studies in which validity or reliability were not supported by a citation were supported by funding from industry or a mix of industry and non-industry sources.

Our study findings are consistent with previous studies that examined psychometric properties of AD measures. Demers and colleagues reviewed the psychometric properties of outcome measures used in AD trials and reported their validity and reliability [99–102]. They reported on three measures for global status, eight measures for function and eight measures for behaviour. Across outcome measures, they found variable evidence of reliability and validity. Of note, they identified that function and behaviour measures lacked evidence of validity and reliability [99–102]. The work by Dermers and colleagues was based on a report in 2000 and identified 26 trials that reported use of cognitive enhancers for AD patients. Our study included a systematic search for randomized controlled trials as well as observational data and did not include any limitations on years of publication. As such, we identified more outcome measures for function, behaviour, and global status and also examined cognition, which was not reported in their previous work.

Locating validity and reliability information for cognition was a challenge. A majority (n = 24) of the valid and reliable scales to measure cognition were published before 2000, suggesting that cognition measures used in AD trials have not changed substantially over time. We observed a high number of cases where a cognition measure was only used in one study, which makes it difficult to make comparisons or draw conclusions across studies. In 2001, the MMSE [12] became a proprietary measure, which means a licensed version had to be purchased before use [103]. Many researchers and clinicians are compelled to use open source tools to assess cognition. The Montreal Cognitive Assessment (MoCA) measure has evidence of validity and but was not used in any of the included studies and it has not been validated for use in as many settings as the MMSE.

Our study has some limitations. First, the charting exercise was conducted on the original systematic review search (December 2011) rather than updated literature search. We conducted the charting exercise to gain insight on which measures were used in studies to assess outcomes; as such, we did not update the charting exercise as outcome measures were already selected. Second, we followed a three-step process for identifying validity and reliability information rather than completing a systematic search for each outcome measure. Given limited resources, we felt our three-step approach was feasible and yielded information needed to select outcome measures for our systematic review. Third, we did not assess the methodological quality of the validity and reliability information and merely categorized whether there was reported evidence of validity or reliability based on how authors reported the information. Lastly, we did not search grey literature sources to obtain validity and reliability evidence, given limited resources.

## Conclusions

Our paper highlights the variability in the reporting of outcome measures used in AD studies. We identified multiple outcome measures reported in the primary studies; many of these were used only once in the primary studies that were included in our systematic review. The large number of measures used in studies makes it difficult to synthesize the evidence. Cataloguing and assessing validity and reliability of each outcome measure in studies can be resource intensive; using core outcome sets (i.e., agreed upon outcomes and measures used in a particular discipline) in systematic reviews is recommended as this may streamline outcome selection [6]. In lieu of core outcome sets, researchers need to balance frequency of reported outcome measures, their respective validity and reliability, and preferences of knowledge users.

## Supporting information

S1 Table Validity and Reliability Definitions. (PDF)

S2 Table Frequency of Cognitive Outcome Measures (n = 57). (PDF)

S3 Table Frequency of Functional Outcome Measures (n = 21). (PDF)

S4 Table Frequency of Behavioural Outcome Measures (n = 13). (PDF)

S5 Table Frequency of Global Status Outcome Measures (n = 10). (PDF)

S6 Table Missing References and Sources of Funding. (PDF)
