# Supplementary material for: Variability in the validity and reliability of outcome measures identified in a systematic review to assess treatment efficacy of cognitive enhancers for Alzheimer’s Dementia
Source: PLoS One. 2019 Apr 18;14(4):e0215225. doi: 10.1371/journal.pone.0215225 (PMC6472754; doi:10.1371/journal.pone.0215225)
Supplement: S1 Table — (PDF) [file pone.0215225.s001.pdf]

S1 Table. Validity and Reliability Definitions

		Type	Definition
Construct	Translational	Face/Content	Based on the consensus or judgement of experts, the instrument or scale appears to measure or capture the complete range of attributes of the construct of interest.
	Criterion	Concurrent	The extent to which scores on a scale correlate with some other scale that measures the same construct and are tested at the same time.
		Predictive	The extent to which scores on a scale correlate with some other scale that measures the same construct and the results of which are not known until some future time.
		Convergent	The extent to which different scales measuring the same construct are related.
		Discriminant	The extent to which different scales measuring different constructs are related
Reliability		Internal consistency	Extent to which performance on one item on a scale is a good indicator for performance on another item in the scale
		Test-retest	The extent to which the scoring or performance on an instrument or scale is stable over time.
		Inter-rater reliability	The degree of agreement between administrators.

Source: Trochim W (2001). The Research Methods Knowledge Base (2nd Ed.). Cincinnati, OH. Atomic Dog Publishing
