# Supplementary material for: Variability in the validity and reliability of outcome measures identified in a systematic review to assess treatment efficacy of cognitive enhancers for Alzheimer’s Dementia
Source: PLoS One. 2019 Apr 18;14(4):e0215225. doi: 10.1371/journal.pone.0215225 (PMC6472754; doi:10.1371/journal.pone.0215225)
Supplement: S2 Table — (PDF) [file pone.0215225.s002.pdf]

S2 Table. Frequency of Cognitive Outcome Measures (n=57)

Name	Total
Mini Mental State Exam (MMSE)	80
Alzheimer's Disease Assessment Scale- Cognitive subscale (ADAS-Cog)	61
Severe Impairment Battery (SIB)	13
Global Deterioration Scale (GDS)	10
Clinical Dementia Rating Scale (CDR)	7
Clinical Dementia Rating Scale – Sum of Boxes (CDR-SB)	7
Trail Making Test (TMT)	5
Clock Drawing Test (CDT)	4
Digit Span Test (DS)	4
Digit Symbols Test (DST)	4
Raven Colored Matrices (RCM)	3
Stroop Test (ST)	3
Stockholm Gerontology Research Center test (SGRC)	3
Verbal Fluency (VF)	3
Blessed Dementia Scale (BDS)	2
Clock Recognition (C-Recog)	2
Rey Auditory Verbal Learning Test (RAVLT)	2
Rey-Osterrieth Complex Figure Copy (ROCF- copy)	2
Story Recall (SR)	2
Trail Making Test A (TMT-A)	2
Stockholm Gerontology Research Center test- D-prime value (SGRC D-prime)	2
Word Paradigm- free recall (WP-FR)	2
Alzheimer's Disease Assessment Scale (ADAS)	1
Alzheimer's Disease Assessment Scale - Orientation Test (ADAS-OT)	1
American Speech-Language Hearing Association- Functional Assessment of Communication Skills for Adults Basic needs and social communication subscales (ASHA-FACS)	1
Cambridge Cognitive Examination (CAMCOG)	1
Cambridge Automated Neuropsychiatric Test Assessment Battery (CANTAB)	1
Cognitive Drug Research Test Battery (CDRTB)	1
Consortium to Establish a Registry for Alzheimer's Disease –cog subscale (CERAD-cog)	1
Category Fluency Test (CFT)	1
Computerized Memory Battery Test (CMBT)	1
Controlled Oral Word Association Test (COWAT)	1
Delayed Word Recall (DWR)	1

Forced Delayed Recognition (FDR)	1
Frontal Assessment Battery (FAB)	1
Functional Linguistic Communication Inventory (FLCI)	1
Groton Maze Learning Task (GMLT)	1
Immediate Visual Memory (IVM)	1
Multiple Feature Target Cancellation (MFTC)	1
Non-Demanding Test of Visual Attention (NDTVA)	1
NYU stories test delayed recognition subscale (NYU-ST-DRecog)	1
Oral Production Test	1
Reading and Setting a Clock Test (RSCT)	1
Rey-Osterrieth complex figure recall (ROCF-recall)	1
Syndrom Kurz test (SKT)	1
Serial Reaction Time Task (SRTT)	1
Spatial Span (SS)	1
Test of Constructional Praxis (TCP)	1
Temporal Rule Induction (TRI)	1
Token Test (TT)	1
Visual Motor Gestalt Test (Bender) (VMGT)	1
Wechsler Adult Intelligence Scale (WAIS)	1
Word Fluency (WF)	1
Word Learning (WL)	1
Wechsler Logical Memory Test (WLMT)	1
Wechsler Memory Scale (WMS)	1
Zahlen–Verbindungs Test (ZVT)	1
