# Supplementary material for: Variability in the validity and reliability of outcome measures identified in a systematic review to assess treatment efficacy of cognitive enhancers for Alzheimer’s Dementia
Source: PLoS One. 2019 Apr 18;14(4):e0215225. doi: 10.1371/journal.pone.0215225 (PMC6472754; doi:10.1371/journal.pone.0215225)
Supplement: S3 Table — (PDF) [file pone.0215225.s003.pdf]

## S3 Table. Frequency of Functional Outcome Measures (n=21)

| Measure | Total |
|---|---|
| Physical Self-Maintenance/Activities of Daily Living (ADL) | 19 |
| Alzheimer's Disease Cooperative Studies–Activities of Daily Living Inventory (ADCS-ADL) | 14 |
| Disability Assessment for Dementia (DAD) | 14 |
| Instrumental Activities of Daily Living (IADL) | 10 |
| Progressive Deterioration Scale (PDS) | 8 |
| Alzheimer's Disease Cooperative Studies Activities of Daily Living Severe impairment subscale (ADCS-ADL-severe) | 6 |
| Functional Assessment Screening Tool (FAST) | 6 |
| Barthel Index (BI) | 2 |
| Caregiver-rated Modified Crichton Scale (CMCS) | 2 |
| Mental Function Impairment Scale (MENFIS) | 2 |
| Nurses Observation Scale for Geriatric Patients (NOSGER) | 2 |
| Activity & Affect Indicators of Quality of Life (AAIQOL) | 1 |
| Alzheimer's Disease Functional Assessment and Change Scale (ADFACS) | 1 |
| Bristol Activities of Daily Living (BADL) | 1 |
| Caregiver Burden Questionnaire (CBQ) | 1 |
| Caregiver Perceived Burden Questionnaire (CPBQ) | 1 |
| Functional Rating Scale (FRS) | 1 |
| Global Assessment of Functioning Scale (GAFS) | 1 |
| Goal Attainment Scale (GAS) | 1 |
| Interview for Deterioration in Daily Living Activities in Dementia (IDDD) | 1 |
| Zarit Burden Interview (ZBI) | 1 |
