# Supplementary material for: Variability in the validity and reliability of outcome measures identified in a systematic review to assess treatment efficacy of cognitive enhancers for Alzheimer’s Dementia
Source: PLoS One. 2019 Apr 18;14(4):e0215225. doi: 10.1371/journal.pone.0215225 (PMC6472754; doi:10.1371/journal.pone.0215225)
Supplement: S4 Table — (PDF) [file pone.0215225.s004.pdf]

S4 Table. Frequency of Behavioural Outcome Measures (n=13)

**Measure**	**Total**
Neuropsychiatric Inventory (NPI)	36
Behavioural Pathology in Alzheimer's Disease Rating Scale (BEHAVE-AD)	5
Cohen-Mansfield Agitation Inventory (CMAI)	5
Neuropsychiatric Inventory – Caregiver distress scale (NPI-CDS)	4
Behavioural Rating Scale for Geriatric Patients (BRS)	3
Apathy Scale (AS)	1
Brief Neuropsychiatric Inventory Scale (b-NPI)	1
Brief Psychiatric Rating Scale (b-PRS)	1
Caregiver-Administered-Neuropsychiatric Inventory (CA-NPI)	1
Crichton Geriatric Rating Scale (CGRS)	1
Geriatric Depression Scale (GDS)	1
Neuropsychiatric Inventory –Nursing Home version (NPI-NH)	1
Pittsburgh Agitation Scale (PAS)	1
