# Supplementary material for: Variability in the validity and reliability of outcome measures identified in a systematic review to assess treatment efficacy of cognitive enhancers for Alzheimer’s Dementia
Source: PLoS One. 2019 Apr 18;14(4):e0215225. doi: 10.1371/journal.pone.0215225 (PMC6472754; doi:10.1371/journal.pone.0215225)
Supplement: S5 Table — (PDF) [file pone.0215225.s005.pdf]

S5 Table. Frequency of Global Status Outcome Measures (n=10)

Measure	Total
Clinician Interview-Based Impression of Change plus caregiver input (CIBIC-plus)	35
Clinical Global Impression of Change (CGIC)	10
Clinical Global Impression of Improvement (CGI-I)	3
Alzheimer's Disease Cooperative Studies – Clinician Global Impressions of Change (ADCS-CGIC)	2
Caregiver Stress Scale (CSS)	2
Gottfries-Bråne-Steen Scale (GBS)	2
Caregiver-rated Global Impression (CRGI)	1
Clinical Global Impression of Change - severe subscale (CGIC-severe)	1
Patient Global Assessment Scale (PGA)	1
Screen for Caregiver Burden (SCB)	1
