# Supplementary material for: Variability in the validity and reliability of outcome measures identified in a systematic review to assess treatment efficacy of cognitive enhancers for Alzheimer’s Dementia
Source: PLoS One. 2019 Apr 18;14(4):e0215225. doi: 10.1371/journal.pone.0215225 (PMC6472754; doi:10.1371/journal.pone.0215225)
Supplement: S6 Table — (PDF) [file pone.0215225.s006.pdf]

S6 Table. Missing References and Sources of Funding

Measure	Funding
Patient Global Assessment (PGA)[1]	Industry sponsored
Mental Function Impairment Scale (MENFIS)[2, 3]	Industry sponsored
Caregiver burden questionnaire[4]	Industry sponsored
Digit Span[5-8]	Non-industry sponsored; Mixed funding (industry and non-industry sponsored); not reported, industry sponsored; industry sponsored
Digit Symbols test[9-11]	Non-industry sponsored; non-industry sponsored; Mixed funding (industry and non-industry sponsored)
Stockholm Gerontology Research Center Test[9, 10, 12]	Mixed funding (industry and non-industry sponsored); Mixed funding (industry and non-industry sponsored); Mixed funding (industry and non-industry sponsored)
Stockholm D-prime[9, 11]	Mixed funding (industry and non-industry sponsored); Mixed funding (industry and non-industry sponsored)
Stroop Test[13] [7, 14]	Not reported; not reported, not reported; industry sponsored
Clock recognition[10, 12]	Mixed funding (industry and non-industry sponsored); Mixed funding (industry and non-industry sponsored)
Verbal Fluency[5, 6]	Non-industry sponsored; Mixed funding (industry and non-industry sponsored)
CANTAB[15]	Mixed funding (industry and non-industry sponsored)
Category Fluency Test[14]	Industry sponsored
Cognitive Drug research test battery[16]	Industry sponsored
Computerized memory battery test [1]	Industry sponsored
Forced delayed recognition[5]	Non-industry sponsored
Immediate visual memory[5]	Non-industry sponsored
Multiple feature cancellation test[5]	Non-industry sponsored
Non-demanding test of visual attention[5]	Non-industry sponsored

NYU stories test delayed recognition subscale [17]	Non-industry sponsored
Reading and setting a clock[11]	Mixed funding (industry and non-industry sponsored)
Serial reaction test[18]	Mixed funding (industry and non-industry sponsored)
Spatial span[6]	Mixed funding (industry and non-industry sponsored)
Temporal rule induction[5]	Non-industry sponsored
Test of constructional praxis[5]	Non-industry sponsored
Token Test[6]	Mixed funding (industry and non-industry sponsored)
Visual motor gestalt[19]	Not reported
WAIS (performance and verbal IQ)[20]	Not reported
Verbal fluency [17]	Non-industry sponsored
Word Learning [7]	Not reported

References

[1] Seltzer B, Zolnouni P, Nunez M, Goldman R, Kumar D, Ieni J, et al. Efficacy of donepezil in early-stage Alzheimer disease: a randomized placebo-controlled trial. Arch Neurol. 2004;61:1852-6.

[2] Homma A. NR, Ishii T., Hasegawa K. Development of a new rating scale for dementia in the elderly: Mental function impairment scale (MENFIS). Japanese Journal of Geriatric Psychiatry. 1991;2:1217-22.

[3] Homma A, Takeda M, Imai Y, Udaka F, Hasegawa K, Kameyama M, et al. Clinical efficacy and safety of donepezil on cognitive and global function in patients with Alzheimer's disease. A

24-week, multicenter, double-blind, placebo-controlled study in Japan. E2020 Study Group. Dement Geriatr Cogn Disord. 2000;11:299-313.
[4] Black SE, Doody R, Li H, McRae T, Jambor KM, Xu Y, et al. Donepezil preserves cognition and global function in patients with severe Alzheimer disease. Neurology. 2007;69:459-69.
[5] Bizzarro A, Marra C, Acciarri A, Valenza A, Tiziano FD, Brahe C, et al. Apolipoprotein E epsilon4 allele differentiates the clinical response to donepezil in Alzheimer's disease. Dement Geriatr Cogn Disord. 2005;20:254-61.
[6] Lorenzi M, Beltramello A, Mercuri NB, Canu E, Zoccatelli G, Pizzini FB, et al. Effect of memantine on resting state default mode network activity in Alzheimer's disease. Drugs Aging. 2011;28:205-17.
[7] Nakano S, Asada T, Matsuda H, Uno M, Takasaki M. Donepezil hydrochloride preserves regional cerebral blood flow in patients with Alzheimer's disease. J Nucl Med. 2001;42:1441-5.
[8] Forette F, Anand R, Gharabawi G. A phase II study in patients with Alzheimer's disease to assess the preliminary efficacy and maximum tolerated dose of rivastigmine (Exelon). Eur J Neurol. 1999;6:423-9.
[9] Darreh-Shori T, Kadir A, Almkvist O, Grut M, Wall A, Blomquist G, et al. Inhibition of acetylcholinesterase in CSF versus brain assessed by 11C-PMP PET in AD patients treated with galantamine. Neurobiol Aging. 2008;29:168-84.
[10] Kadir A, Darreh-Shori T, Almkvist O, Wall A, Grut M, Strandberg B, et al. PET imaging of the in vivo brain acetylcholinesterase activity and nicotine binding in galantamine-treated patients with AD. Neurobiol Aging. 2008;29:1204-17.

[11] Stefanova E, Wall A, Almkvist O, Nilsson A, Forsberg A, Langstrom B, et al. Longitudinal PET evaluation of cerebral glucose metabolism in rivastigmine treated patients with mild Alzheimer's disease. J Neural Transm (Vienna). 2006;113:205-18.
[12] Keller C, Kadir A, Forsberg A, Porras O, Nordberg A. Long-term effects of galantamine treatment on brain functional activities as measured by PET in Alzheimer's disease patients. J Alzheimers Dis. 2011;24:109-23.
[13] Borkowska A, Ziolkowska-Kochan M, Rybakowski JK. One-year treatment of Alzheimer's disease with acetylcholinesterase inhibitors: improvement on ADAS-cog and TMT A, no change or worsening on other tests. Hum Psychopharmacol. 2005;20:409-14.
[14] Wilkinson D, Fox NC, Barkhof F, Phul R, Lemming O, Scheltens P. Memantine and brain atrophy in Alzheimer's disease: a 1-year randomized controlled trial. J Alzheimers Dis. 2012;29:459-69.
[15] Nordberg A, Darreh-Shori T, Peskind E, Soininen H, Mousavi M, Eagle G, et al. Different cholinesterase inhibitor effects on CSF cholinesterases in Alzheimer patients. Curr Alzheimer Res. 2009;6:4-14.
[16] Frolich L, Ashwood T, Nilsson J, Eckerwall G, Sirocco I. Effects of AZD3480 on cognition in patients with mild-to-moderate Alzheimer's disease: a phase IIb dose-finding study. J Alzheimers Dis. 2011;24:363-74.
[17] Greenberg SM, Tennis MK, Brown LB, Gomez-Isla T, Hayden DL, Schoenfeld DA, et al. Donepezil therapy in clinical practice: a randomized crossover study. Arch Neurol. 2000;57:94-9.

[18] Winstein CJ, Bentzen KR, Boyd L, Schneider LS. Does the cholinesterase inhibitor, donepezil, benefit both declarative and non-declarative processes in mild to moderate Alzheimer's disease? Curr Alzheimer Res. 2007;4:273-6.

[19] Abolfazli R, Ghazanshahi, S., Nzaeman, M. Effects of 6 months of treatment with Donepezil, and Rivastigmine on results of neuropsychologucal tests of MMSE, NPI, Clock and Bender in patients with Alzheimer's Disease. Acta Medica Iranica. 2008;46:99-104.

[20] Thomas A, Iacono D, Bonanni L, D'Andreamatteo G, Onofrj M. Donepezil, rivastigmine, and vitamin E in Alzheimer disease: a combined P300 event-related potentials/neuropsychologic evaluation over 6 months. Clin Neuropharmacol. 2001;24:31-42.
